# Prognostic Role of Functional SYNTAX Score Based on Quantitative Flow Ratio

**DOI:** 10.3390/biomedicines12112437

**Published:** 2024-10-24

**Authors:** Dimitrios Terentes-Printzios, Konstantia-Paraskevi Gkini, Dimitrios Oikonomou, Vasiliki Gardikioti, Konstantinos Aznaouridis, Ioanna Dima, Konstantinos Tsioufis, Charalambos Vlachopoulos

**Affiliations:** First Department of Cardiology, Hippokration Hospital, Medical School, National and Kapodistrian University of Athens, 114 Vasilissis Sofias St., 11527 Athens, Greece; gk.konstantia@gmail.com (K.-P.G.); jimoik4@hotmail.com (D.O.); conazna@yahoo.com (K.A.); ioadim2006@yahoo.gr (I.D.); kptsioufis@gmail.com (K.T.); cvlachop@otenet.gr (C.V.)

**Keywords:** QFR, FSSQFR, prognosis, coronary angiography, SYNTAX, mortality

## Abstract

Background/Objectives: The quantitative flow ratio (QFR)-based functional Synergy Between Percutaneous Coronary Intervention with Taxus and Cardiac Surgery (SYNTAX) score (FSSQFR) combines coronary arteries’ anatomy and physiology. Methods: We performed an offline FSSQFR calculation in all-comers undergoing coronary angiography in a single center. Based on the tertiles of SYNTAX Score (SS), patients were divided into low-, intermediate-, and high-risk groups with the following cut-offs: SS/FSSQFR < 13, SS/FSSQFR: 13–21, and SS/FSSQFR: >21. The primary endpoint was the predictive value of the FSSQFR of the composite endpoint of all-cause death, myocardial infarction, ischemia-driven revascularization, hospitalization for heart failure, and life-threatening arrhythmias after the follow-up period. Results: This study included 410 patients. SS and FSSQFR were measured for all patients. After calculating FSSQFR, the risk stratification changed in 11% of the study population; more specifically, 26.8, 32.7, and 40.5% of patients were classified as high-, intermediate-, and low-risk, respectively. After a median 30.2 (25.7–33.7) months follow-up period, we recorded 85 events of the primary outcome. The high-risk FSSQFR group compared to the low-risk group had a significantly higher rate of the primary composite outcome (HR: 1.95, 95% CI 1.33–3.34, *p* = 0.016). Conclusions: In our study, patients classified as the high-risk FSSQFR group had a significantly higher rate of cardiovascular adverse events.

## 1. Introduction

The Synergy Between Percutaneous Coronary Intervention with Taxus and Cardiac Surgery (SYNTAX) Score (SS) is an angiographic grading tool developed to assess the anatomically coronary complexity in patients with coronary artery disease. It has been shown to predict the risk for future adverse events in patients with left main (LM) or multivessel disease [1]. Based on the tertiles of anatomical SS, patients were divided into low-, intermediate-, or high-risk groups. This classification suggested the optimal treatment strategy for each risk group, including coronary artery bypass (CABG) or percutaneous coronary intervention (PCI) [2,3,4,5]. Recent large-scale trials reported that the physiology-guided PCI based on the fractional flow reserve (FFR) value is superior to angiography-only guided PCI regarding clinical outcomes and is thus currently recommended by guidelines for decision-making during PCI [1,6,7,8].

FFR is an invasive method using a pressure wire for estimating the hemodynamic severity of coronary stenosis and is considered the gold-standard method for physiology assessment [9,10]. FFR values ≤ 0.80 suggest ischemia-producing lesions requiring revascularization [11]. The functional SS by summing the score only of hemodynamic significant stenosis as decided by FFR (FSSFFR), changes the risk stratification and predicts more reliably the risk for adverse events after PCI in patients with multivessel coronary artery disease [12]. Despite the described benefit of physiology-guided revascularization, FFR-guided PCI demanded increased time and cost and drug administration, resulting in its underutilization, according to real-world data [13].

Plenty of new software calculating FFR by plain angiography has emerged. The quantitative flow ratio (QFR) combines the computation of fluid dynamics and a 3D anatomical vessel reconstruction based on two angiographical views without the need for coronary vessel instrumentation with a pressure wire and the administration of vasodilator agents. This method has demonstrated good correlation and diagnostic accuracy compared to the FFR [13,14,15]. QFR is recommended for functional assessment of epicardial artery stenosis artery severity during invasive coronary angiography to guide revascularization [16].

Reassessing the anatomical SS by incorporating only ischemia-producing lesions determined by QFR is a refining grading tool that combines the coronary tree’s anatomy and physiology (FSSQFR), lacking the drawbacks of FFR. It has been shown that FSSQFR altered the risk stratification for adverse events and changed the therapy option in patients with LM or multivessel coronary artery disease in the context of a clinical trial [17].

The primary objective of this study was to estimate the predictive value of FSSQFR for the composite endpoint of all-cause death, myocardial infarction (MI), ischemia-driven revascularization, hospitalization for heart failure, and life-threatening arrhythmias after the follow-up period and compare its prognostic role to the SS. Second, we sought to investigate the FSSQFR as a predictor for MACE (including cardiovascular death, MI, and ischemia-driven revascularization) and cardiac death. Finally, after the follow-up period, we investigated the association between FSSQFR and angina symptoms based on the Canadian Cardiovascular Society (CCS) grading system.

## 2. Materials and Methods

### 2.1. Study Design

The investigators considered for enrollment for this study patients who had coronary artery disease, were prospectively enrolled in a large cohort assessing eligibility for proprotein convertase subtilisin–kexin type 9 inhibitors treatment, and underwent coronary angiography in ‘Hippokration’ General Hospital between October 2018 and December 2019 [18]. Eligible patients were those with all three vessels satisfying the requirements for QFR measurement, irrespective of the extent of disease (single-, two-, or three-vessel disease). Although this scoring system has been developed with reference to the SYNTAX trial, comparing PCI with CABG in patients with LM and multivessel coronary artery disease, it is also capable of predicting major cardiovascular events in patients undergoing PCI, suggesting that more complicated lesions result in worst prognosis [19]. Reasons for exclusion were the software incompatibility with the coronary angiography and the presence of at least one non-analyzable QFR vessel. The investigators recorded each specific reason for exclusion.

**Figure 1:**
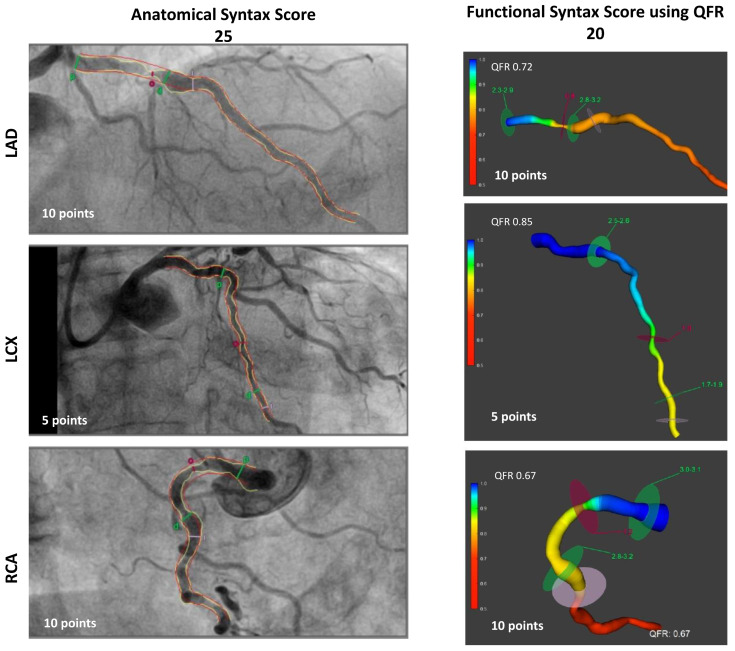
A patient with a 3-vessel disease was classified as high-risk because the anatomical SYNTAX Score yielded 25 points. The same patient was reclassified to the intermediate-risk group based on FSSQFR calculated to 20 points. The functional assessment using QFR revealed that the left circumflex lesions were not hemodynamically significant. FSSQFR: functional SYNTAX Score based on quantitative flow ratio.

In addition to assessing the SS, the FSSQFR was calculated by summing the value from the SYNTAX Score only for ischemia-inducing lesions defined by a QFR ≤ 0.80 (Figure 1).

Patients were divided into three risk categories based on tertiles of SS, and, by the same cut-off points, population was stratified into 3 groups based on FSSQFR: low risk (FSSQFR/SS: <13), intermediate risk (FSSQFR/SS: 13–21), and high risk (FSSQFR/SS: >21). Adopting thresholds that were slightly different from those based on the SYNTAX Score, we aimed to have comparable results to the previous two studies [17,20]). Investigators collected demographic data, cardiovascular risk factors, indications for coronary angiography, and procedural data from the medical records of the study center. The follow-up was carried out by outpatient clinic visits, review of medical records, or telephone calls. This study was conducted following the Declaration of Helsinki, and the study protocol was approved by the Institutional Review Board of the ‘Hippokration’ General Hospital.

### 2.2. Study Population

The eligible population for this study was patients ≥ 18 years of age, regardless of gender, race, and extent of CAD, who underwent coronary angiography for any indication. Patients with CABG history were excluded. In each patient, calculation of the QFR in all three coronary vessels was required. Patients with non-analyzable QFR in any coronary artery were not eligible for this study. Native vessels were inappropriate for QFR analysis if they

1. Had ostial LM disease;
2. Were small in size with reference luminal diameter < 2.0 mm by visual assessment;
3. Presented slow coronary blood flow (TIMI [Thrombolysis In Myocardial Infarction] flow grade 1 or 2);
4. Had <2 projections with isocenter calibration information;
5. Had severe vessel overlap;
6. Had poor angiographic image quality, precluding precise contour delineation.

### 2.3. Coronary Angiography and QFR Computation

Patients underwent conventional coronary angiography and PCI or were referred for CABG based on the discretion of the operator or the Heart Team according to best local practice. Two validated, blinded to the visual estimation of the interventional cardiologist and the therapeutic decision, experienced analysts certified for using the software with the QAngio XA 3D version 1.1 software package (Medis Medical Imaging Systems, Leiden, The Netherlands) performed off-line QFR analysis. To compute QFR, contrast QFR without hyperemic setting along with the frame-counting method was applied. QFR was analyzed from the ostium of the main vessels (left anterior descending, right, and left circumflex coronary arteries) to their distal anatomic part. Major side branches with reference diameter ≥ 2.0 mm were also considered eligible for QFR analysis. In this study, the FSS derived from the QFR (FSSQFR) assessment was calculated by summing the individual points of physiologically significant lesions and excluding physiologically nonsignificant lesions based on the QFR estimation (Figure 1). Of note, the FSSQFR included the respective points from SS calculation in case of a chronic total occlusion. The classic anatomic SS was calculated on-site based on a visual evaluation of significant lesions (diameter stenosis > 50% in vessels diameter 1.5 mm) for all patients eligible for the present study. An online calculator, SYNTAX Score version 2.28 (SYNTAX Score Working Group), was used to derive total and per lesion SS.

### 2.4. Endpoints

The primary endpoint was the composite outcome of all-cause death, MI, ischemia-driven revascularization, hospitalization for heart failure, and life-threatening arrhythmias. The secondary endpoints included the incidence of MACEs (including cardiac death, MI, and ischemia-driven revascularization), cardiac death, and the angina estimation using the CCS grading system after the follow-up period. The investigators used the fourth universal definition of MI [21]. Any revascularization (PCI or CABG) in the presence of angina combined or not with abnormal non-invasive functional diagnostic test results was defined as ischemia-driven revascularization. Sustained ventricular tachycardia (VT), ventricular fibrillation, or torsades de pointes were considered life-threatening arrhythmias. The first adverse event that occurred in any patient was the one reported.

### 2.5. Sample Size Calculation and Statistical Analysis

Based on the hypothesis that the patients with high FSSQFR have two and a half-fold [15] increased risk of presenting the primary endpoint compared to the low FSSQFR in a two-year follow-up period compared to the patients with intermediate or low FSSQFR, we calculated a priori that a sample size of 400 subjects would be sufficient to provide 80% power with an alpha value of 0.05. With a prediction of a possible 2.5% loss of patients at follow-up, we decided to include 410 subjects in total.

Continuous variables were presented either as the mean ± SD or as the median with the interquartile range regarding whether they had normal or no distribution, respectively. Categorical variables were reported as numbers and percentages. We performed group comparisons for categorical variables using the chi-square test or the Fisher exact test, as appropriate, while using a one-way ANOVA test for continuous variables and Krussal–Wallis test for continuous variables non-normally distributed. A 2-sided *p* < 0.05 indicates statistical significance. Receiver-operator characteristic curves analysis with area under the curve (AUC) was used to compare the prediction capability of SS and FSSQFR for the primary composite endpoint during the follow-up period. The risk reclassification from SS to FSSQFR for primary composite endpoint was assessed using net reclassification improvement (NRI) and integrated discrimination improvement. Multivariate Cox regression analysis was used to estimate hazard ratios (HR) and 95% CI. The Cox regression model was created based on the variables that were predictors of the primary outcome with *p*-values ≤ 0.05 in univariate analysis (Table S2). Multivariate binary logistic regression analysis was performed to determine whether the high-risk FSSQFR group was an independent predictor of moderate to severe angina (CCS II-IV). All statistical analyses were performed using the SPSS statistical package for Windows (version 24.0, SPSS Inc., Chicago, IL, USA) and STATA 13.0 SE (Stata Corp., College Station, TX, USA).

## 3. Results

### 3.1. Population

**Figure 2:**
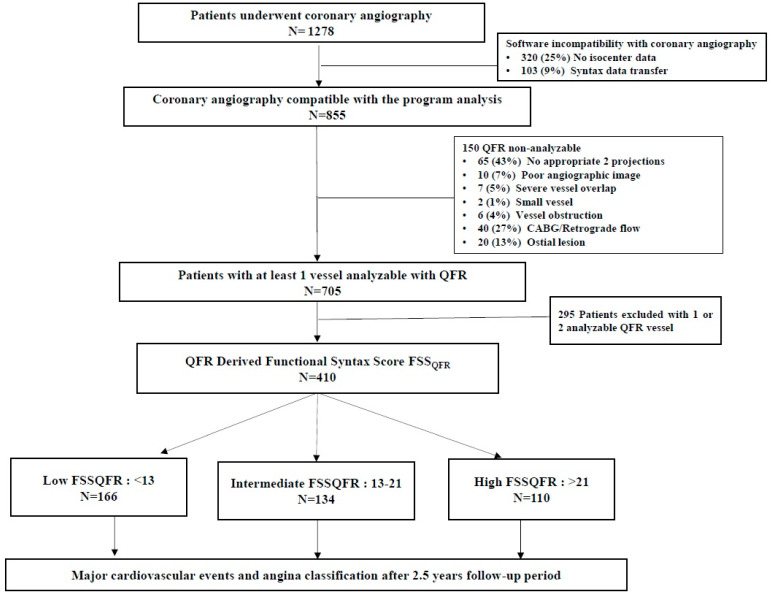
Study flowchart. CABG: coronary artery bypass graft, QFR: quantitative flow ratio, FSSQFR: functional SYNTAX Score based on quantitative flow ratio.

Initially, 1278 patients who underwent coronary angiography were screened for eligibility from June 2018 until September 2019 at our center. Software incompatibility was the exclusion reason for 423 patients. Among the remaining patients, non-analyzable QFR vessels, mainly due to the lack of the two appropriate projections, were the exclusion cause for 150 patients. Also, we excluded 295 patients because they had only one or two analyzable QFR vessels. Finally, 410 patients were eligible for this study (Figure 2, Table S1).

The mean age of the study population was 65.3 ± 11 years, and 82.7% of patients were male. The proportion of the study population with a history of dyslipidemia, hypertension, and smoking was 79.8, 67.7, and 74.1%, respectively. Also, more than half of the population had a history of previous MI. Chronic coronary syndrome was the indication of coronary angiography in most patients (61%). Table 1 summarizes overall patients’ baseline and procedural characteristics.

### 3.2. Risk Stratification

**Figure 3:**
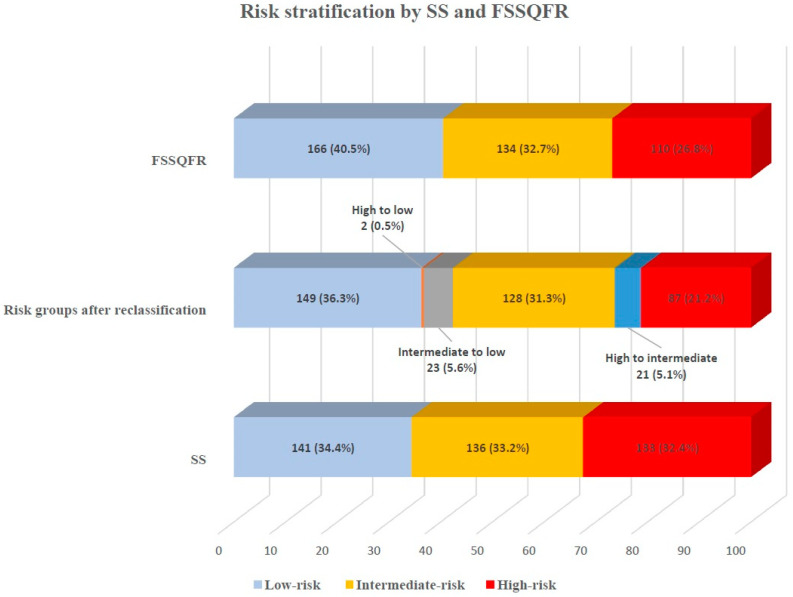
Risk stratification based on SS and FSSQFR. The percentages of the study patients were classified according to SS and FSSQFR. After FSSQFR calculation, 11% of the study population moved from a higher-risk group to a lower-risk group. Abbreviations: QFR: quantitative flow ratio, FSSQFR: functional SYNTAX Score based on quantitative flow ratio.

The investigators calculated the SS and FSS in all included patients. The median values of SS and FSSQFR were 17.0 (IQR: 8.0–23.0) and 15.0 (IQR: 5.0–22.0), respectively. Based on the tertiles of SS, patients were divided into low-, intermediate-, and high-risk groups with the following cut-offs: SS/FSSQFR < 13, SS/FSSQFR: 13–21, and SS/FSSQFR: >21. Specifically, 141 (34.4%) patients were characterized as low-risk, 136 (33.2%) as intermediate-risk, and 133 (32.4%) as high-risk according to SS values. After the FSSQFR calculation, 46 (11%) patients changed from higher to lower risk group. Based on FSSQFR, 166 patients (40.5%), 134 (32.7%), and 110 (26.8%) were classified as low-, intermediate-, and high-risk groups, accordingly (Figure 3).

Baseline and procedural characteristics divided by FSSQFR and SS groups are presented in Table 1 and Table S3. Baseline clinical characteristics, including age, BMI, hypertension, diabetes, dyslipidemia, and smoking, were mainly comparable among the FSSQFR and SS groups except for the history of previous myocardial infarction and heart failure. As expected, the variables showing the complexity of coronary disease, including SS, FSSQFR, total length of stents, stents per patient, and lesion location, were significantly different between the groups of FFSQFR. Baseline and lesion characteristics of reclassified patients based on FFSQFR are presented in Table S4.

### 3.3. Outcomes at Follow-Up

Median follow-up period was 30.2 (IQR: 25.7–33.7) months. During that period, we recorded 85 events of the primary outcome (37 all-cause deaths, 11 MIs, 21 ischemia-driven revascularization, 10 hospitalizations for heart failure, and 6 life-threatening arrhythmias), 60 events of MACEs (28 cardiac deaths, 11 MIs, 21 ischemia-driven revascularization), and 28 cardiac deaths.

***FSSQFR (as a categorical variable):*** The primary composite endpoint was 15.1, 18.7, and 31.8% in the low-, intermediate-, and high-risk FSSQFR groups, respectively. Also, the investigators recorded 9.0, 11.9, and 26.4% MACEs and 2.4, 7.5, and 12.7% cardiac deaths in the corresponding groups (Table 2).

**Figure 4:**
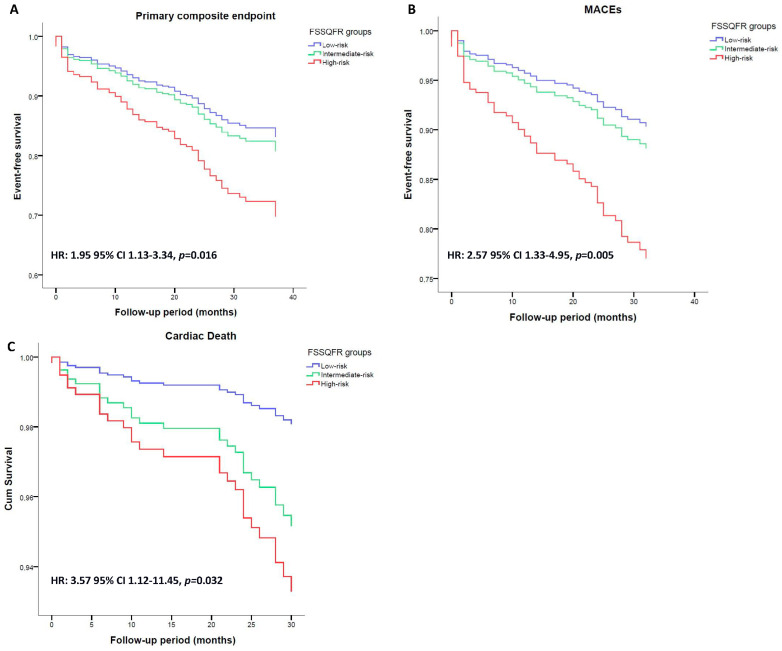
Event-free period by FSSQFR groups. Event-free curves depict (**A**) the composite primary endpoint; (**B**) MACEs; and (**C**) cardiac death. HRs and *p*-value referred to the high-risk group compared to the low-risk group. FSSQFR indicates Synergy between Percutaneous Coronary Intervention with Taxus and Cardiac Surgery score based on quantitative flow ratio and MACEs indicates major cardiac adverse events.

Patients classified as high-risk regarding the FSSQFR presented with a higher risk of all outcomes compared to the low-risk group. Specifically, they had almost 2-fold higher risk of the primary composite outcome and 2.5-fold higher risk of MACE compared to the low-risk group (HR: 1.95 CI 1.33–3.34, *p* = 0.016 Figure 4A and HR: 2.57 CI 1.33–4.95, *p* = 0.005 Figure 4B, respectively). Also, a higher rate of cardiac death was observed in patients stratified as the high-risk FSSQFR group compared to the low-risk group (HR: 3.57 CI 1.12–11.45, *p* = 0.032 Figure 4C).

***SS (as a categorical variable):*** The primary composite endpoint was 15.6, 17.6, and 29.3% in the low-, intermediate-, and high-risk SS groups, respectively (Table S5). Also, we recorded 8.5, 12.5, and 23.3% MACEs and 2.1, 8.1, and 10.5% cardiac deaths in the corresponding groups. The proportion of the study population stratified as the high-risk SS group presented with an almost 2.5-fold higher risk of MACE compared to the low-risk SS group HR: 2.43 95% CI 1.21–4.87, *p* = 0.013 Figure S1B, respectively). Also, patients categorized as high-risk based on SS did not show a statistically significant association for a higher rate neither of the primary composite outcome (HR: 1.72 95% CI 0.995–2.99, *p* = 0.052 Figure S1A) nor of cardiac death (HR: 3.00 95% CI 0.810–11.09, *p* = 0.100 Figure S1C) compared to the low-risk SS group.

**Comparison of FSSQFR versus SS:** There was a strong correlation between FSSQFR and SS values (R^2^ = 0.86, see Figure S2). When comparing the SS to the FSSQFR for the primary endpoint, the two scores have comparable prognostic ability, reclassification, and discrimination (see Supplementary Materials for further analysis, Figure S3). Reclassified patients by the FSS had comparable prognosis to non-reclassified patients (see Table S6).

***FSSQFR (as a continuous variable):*** FSSQFR was an independent predictor of MACE (HR: 1.03 95% CI 1.01–1.06, *p* = 0.01). Patients with higher FSSQFR values presented a non-significant trend to higher risk of primary composite endpoint (HR: 1.02 95% CI 1.00–1.04, *p* = 0.097) and cardiac death (HR: 1.04 95% CI 1.00–1.08, *p* = 0.05) compared to patients with lower values.

**Sensitivity analyses:** Sensitivity analysis was performed by excluding patients managed with CABG. Patients in the high-risk group had a 2.5-fold and 3.5-fold higher risk for primary endpoint and MACEs compared to the low-risk group, respectively, after multivariate Cox analysis (HR: 2.67 95% CI 1.47–4.85, *p* = 0.001 and HR: 3.56 95% CI 1.70–7.48, *p* = 0.01). Also, patients presented in the high-risk group had a five-fold higher risk for cardiac death compared to the low-risk group (HR: 5.14 95% CI 1.27–20.71, *p* = 0.021).

In a second subgroup analysis, we also observed that the results were similar in the ACS to the overall population for the prognostic role of FSSQFR (adjusted HR: 3.62 95% CI 1.21–10.85, *p* = 0.021), whereas the prognostic role of the FSSQFR was t attenuated in the CCS population (adjusted HR: 1.65 95% CI 0.80–3.41, *p* = 0.173) (Table S7).

Patients who were conservatively treated and classified as high-risk had almost a five-fold higher risk for the primary composite outcome compared to the low-risk group (adjusted HR 4.76 95% CI 1.28–17.53, *p* = 0.020). Patients who were treated with PCI and were classified as high-risk FSSQFR had more than a two-fold higher risk for the primary endpoint compared to the low-risk patients (adjusted HR 2.28 95% CI 1.09–4.77, *p* = 0.028).

### 3.4. Angina at Follow-Up

During the follow-up period, 37 patients died. Of the remaining population (N = 373) 82.6% presented without angina or CCS grade I, while 14.7 and 2.7% suffered from angina CCS grade II and III, correspondingly. No patient reported angina CCS grade IV. Patients classified as high-risk FSSQFR group presented more often with moderate to severe angina (CSS II or III) independently of the treatment strategy compared to low-risk FSSQFR group (adjusted OR: 6.99 95% CI 2.94–16.63, *p* < 0.001).

**Figure 5:**
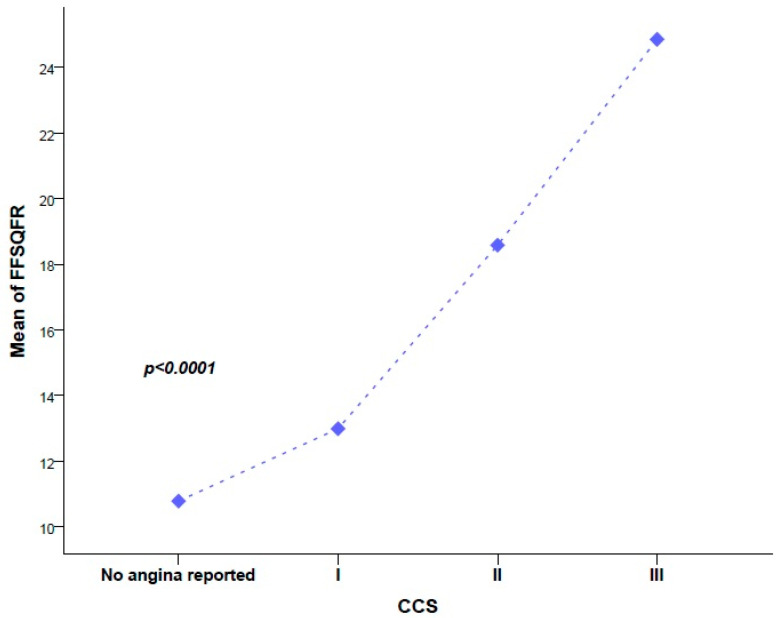
Patients with higher values of FFSQFR had higher grading scores of angina pectoris by the Canadian Society Classification (CCS) system.

Patients complaining of angina CCS grade III had mean FSSQFR = 24.9, while patients with angina CCS grade II or I had lower mean FSSQFR values of 18.6 and 13.0, respectively. Patients without angina symptoms had the lowest mean FSSQFR. Figure 5 shows that the higher the value of baseline FSSQFR, the more severe the angina at follow-up (*p* < 0.0001).

## 4. Discussion

This study presents data from a large real-world cohort with long follow-ups in all-comers undergoing coronary angiography to investigate the prognostic value of FSSQFR. The main findings are:

1. Patients classified in the high-risk FSSQFR group presented with significantly higher rates of primary composite endpoint, MACEs, and cardiac death compared to the low-risk group during the follow-up period with a predictive role that was comparable or slightly better to the SS.
2. FSSQFR was an independent predictor of MACEs.
3. A total of 11% of the study population changed the risk stratification group after the FSSQFR calculation.
4. Patients stratified in the high-risk FSSQFR group presented eight-fold more often with severe angina (CSS II or III) independently of the treatment strategy compared to the low-risk FSSQFR group.

Nam et al. developed the first functional SS based on FFR, combining physiologic and anatomical information in patients with multivessel coronary artery disease. FSSFFR integrated the anatomical SS and FFR by summing the SS only in vessels with ischemia-producing lesions, FFR ≤ 0.8 [12]. Although the FSSFFR altered the risk stratification and predicted the risk for adverse events after PCI more reliably, there were specific drawbacks to this tool. FSSFFR was characterized by all the limitations of FFR and precisely increased operation time and cost, possible complications because of the instrumentation of coronary arteries and drug administration, which ended up with the underutilization of this method in the real world [13]. FSSQFR overcame the aforementioned limitations of FFR. Preliminary prospective trial data indicate that FSSQFR and FSSFFR are highly correlated in patients with two- or three-vessel disease. The FSSQFR requires less time and increases the possibility of physiology decision-making for optimal revascularization [22]. In the present study, the availability of QFR measurements of the entire coronary tree was 32.1% of patients. The analyzable QFR in all three coronary vessels ranged from 28.2 to 54.7% in previous retrospective studies [17,20], suggesting that our study shows similar rates of acceptable QFR analyses to previous studies. However, we need to stress that the rates of analyzable QFR in all three vessels are much higher in prospective studies [23].

A previous large-scale study with a similar design to our research included only patients with a multi-vessel coronary artery disease or LM disease that underwent coronary angiography and investigated the predictive value of FSSQFR for cardiac adverse events. This study validated the FSSQFR on prognostication and discriminant improvement of FSSQFR compared to SS in the described population after a two-year follow-up period. Specifically, the high-risk FSSQFR group had 26.4% MACEs, and this study showed that the FSSQFR was an independent predictor for MACEs [17]. Another study with a smaller population (138 patients) investigated the applicability and feasibility of the FSSQFR in patients with a three-vessel disease. This study showed that FSSQFR could have a better discriminant ability than SS in predicting patient-oriented composite outcomes [20]. Another recent study demonstrated that angiography-derived SS was an independent factor of incident ventricular tachycardia or ventricular fibrillation after acute MI [24]. Although in our study comparing the SS to the FSSQFR for the primary endpoint, the two scores had comparable prognostic ability, reclassification, and discrimination, this was the first study to include all-comers patients who underwent coronary angiography. However, patients in the high-risk SS group had a similar risk for the primary composite outcome compared to the low-risk SS group. On the contrary, patients stratified as high-risk based on FSSQFR had an almost 2-fold higher risk for presenting the primary composite endpoint, a 2.5-fold higher risk of MACEs, and a 3.5-fold higher risk for cardiac death than patients in the low-risk FSSQFR group after a 2.5-year follow-up period, implying a slightly better predictive role of FSSQFR compared to SS.

Previous studies described that the portion of patients who were reclassified after the assessment of FSSQFR ranged from 16 to 26.1% [17,20]. A total of 11% of our study’s population moved from the higher to the lower-risk groups. Zhang et al. described that after the re-stratification, 6% of patients who moved from the high-risk group to the intermediate- or low-risk group had PCI as an alternative therapeutic choice beyond the CABG, suggesting that FSSQFR was a valuable tool for better guidance in the revascularization type [17]. This is in agreement with our findings where 11% of our study population was reclassified from high- to intermediate- or low-risk groups, confirming the validity of our results. However, prospective studies are needed to further validate the role of FSSQFR as a tool to guide decision-making in CAD.

Regarding previous studies, approximately 30% of patients after an angiography successfully PCI presented with a recurrence of angina symptoms that influence both the mental and physical health of these patients associated with several patients and procedural characteristics [25,26]. Several mechanisms, both functional and structural, impact the possibility of angina recurrence, including microvascular dysfunction, incomplete revascularization, vasospasm, and complications related to stent implantation [27]. The FAME II study described that revascularization based on the hemodynamic significance of the lesion based on FFR measurements had reduced the rate of the composite endpoint of death, nonfatal MI, and repeat revascularization at one year [6]. FSSQFR, as described previously, is a score taking into consideration only the functionally significant lesions based on QFR. To the best of our knowledge, this is the first study that sought to investigate the FSSQFR as a predictor for angina symptoms. Approximately 82.6% of the study population reported no angina or angina CCS grade I after the follow-up period. Of the remaining patients, 14.7 and 2.7% suffered from angina CCS grade II and III, respectively. Patients in the high-risk FSSQFR group had an almost seven-fold higher risk of suffering from severe angina compared to the low-risk group after multivariate regression analysis independently from the treatment strategy. Worse prognosis and angina severity in patients with high values of FSSQFR despite revascularization might be attributed to incomplete revascularization. This notion is further supported by the prognostic role of high values of residual FSSQFR in our study population. At present, a randomized controlled trial is ongoing to investigate the impact of QFR measurements on anginal symptoms (https://www.clinicaltrials.gov, accessed on 22 March 2021); unique identifier: NCT04808310), which will further elucidate the possible role of QFR use in the improvement of angina [28].

### 4.1. Clinical Implications

The present study validated in all-comers the impact of FSSQFR on the prognosis of cardiovascular events and angina compared with SS among patients with complex CAD (multivessel CAD) but also with single- or two-vessel disease. Our findings suggest that the use of FSSQFR could potentially provide clinical benefits in clinical management. Reduced implementation of FFR has been mainly attributed to the increased operational time and cost, the requirement of an extra pressure wire, and technical aspects as well, such as pressure wire drift and damping. Assessment of QFR is associated with lower cost, shorter assessment time, no risk of wire injury, and similar accuracy to FFR. All these advantages may help promote the concepts of functional assessment in everyday clinical practice and FSSQFR for decision-making in such clinical scenarios.

### 4.2. Strengths and Limitations

The main strength of our study was the inclusion of all-comers representing a real-world cohort of patients undergoing coronary angiography. Also, the long follow-up of these patients as well as the investigation of angina at follow-up provides more insights in the management of these patients. To the best of our knowledge, our study provides the longest follow-up to date of such studies. Nevertheless, several limitations should be mentioned. Firstly, the present study had a retrospective design. Due to the study’s retrospective design and the requirement for three vessels to be analyzable with QFR to estimate FSSQFR, the exclusion rate was relatively high, and 32.1% of the initial population was analyzed (Figure 2, Table S1). Initially, 1278 patients who underwent coronary angiography were screened for eligibility. Software incompatibility was the exclusion reason for 423 patients. Among the remaining patients, non-analyzable QFR vessels, mainly due to the lack of the two appropriate projections, were the exclusion cause for 150 patients. Also, we excluded 295 patients because they had only one or two analyzable QFR vessels. Finally, 410 patients were eligible for this study. This percentage of excluded patients is high [17,20] and potentially could have implications for the interpretation of our results. Therefore, prospective studies with lower exclusion rates are warranted to confirm our results. The size of the study population was decided based on the incidence of the primary outcome, and thus any findings on secondary outcomes, although confirmatory of FSSQFR’s prognostic role, should be considered hypothesis-generating. Secondly, we calculated SS and FSSQFR in all patients who underwent coronary angiography independently from the extent of coronary artery disease. Based on similar previous studies [17,20], patients admitted with acute coronary syndromes were included in our study. Although the use of coronary physiology in the acute setting is considered unreliable, this is less observed in angiography-derived indices as also confirmed by the fact that FSSQFR in our study is also predictive of events in patients with ACS. Recent data described QFR as a valuable tool for the physiological assessment of patients with MI [29].

## 5. Conclusions

FSSQFR, a scoring system combining the anatomy and physiology of coronary arteries, presents applicability in predicting the prognosis in patients undergoing coronary angiography, which is a significant prognostic factor for adverse events. It was calculable in one-third of the study population. After comparing FSSQFR with SS for the primary outcome, FSSQFR was comparable and, in some cases, slightly better than SS. Finally, there was discordance between the two scores allowing for the reclassification of approximately 11% of the study population by downgrading their risk and thus indicating possible alternative treatment strategies in some patients with overestimated anatomical severity.

## Figures and Tables

**Table 1:** Patient baseline characteristics overall and by FSSQFR Groups.

	Overall (N = 410)	Low FSS (N = 166)	Intermediate FSS (N = 134)	High FSS (N = 110)	*p*-Value
Clinical					
Age, year	65.3 ± 11	65.0 ± 10.8	64.8 ± 11.5	66.4 ± 10.8	0.449
Male	340 (82.9)	122 (73.5)	119 (88.8)	99 (90.0)	<0.0001
Body mass index, kg/m^2^	27.7 ± 4.2	28.0 ± 4.0	27.6 ± 4.0	27.5 ± 4.8	0.632
Hypertension	277 (67.6)	116 (69.9)	91 (67.9)	70 (63.6)	0.552
Dyslipidemia	327 (79.8)	132 (79.5)	107 (79.9)	88 (80.0)	0.995
Smoking	304 (74.1)	123 (74.1)	96 (71.6)	85 (77.3)	0.607
Diabetes mellitus	113 (27.6)	43 (25.9)	34 (25.4)	36 (32.7)	0.364
Positive family history	36 (8.8)	12 (7.2)	12 (9.0)	12 (10.9)	0.569
Coronary artery disease	133 (32.4)	48 (28.9)	43 (32.1)	42 (38.2)	0.272
Previous myocardial infarction	216 (52.7)	66 (39.8)	81 (60.4)	69 (62.7)	<0.0001
Previous PCI	97 (23.7)	40 (24.1)	31 (23.1)	26 (23.6)	0.981
Previous stroke	26 (6.3)	8 (4.8)	8 (6.0)	10 (9.1)	0.354
Peripheral arterial disease	21 (5.1)	10 (6.0)	5 (3.7)	6 (5.5)	0.658
Heart Failure	47 (11.5)	9 (5.4)	18 (13.4)	20 (18.2)	0.003
Ejection Fraction, %	49.0 ± 9.2	53.3 ± 7.8	48.3 ± 8.9	43.7 ± 8.5	<0.0001
Clinical presentation					<0.0001
STEMI	75 (18.3)	15 (9.0)	26 (19.4)	34 (30.9)	
NSTEMI	65 (15.9)	17 (10.2)	27 (20.1)	21 (19.1)	
Unstable angina	20 (4.9)	13 (7.8)	3 (2.2)	4 (3.6)	
CCS	250 (61)	121 (72.9)	78 (58.2)	51 (46.4)	
Angiographic					
Baseline SYNTAX Score	17.0 (8.0–23.0)	8.0 (6.0–12.0)	17 (15.0–19.0)	25 (23.0–29.0)	<0.0001
Lesion location					
Left main artery	24 (5.9)	1 (0.6)	4 (3.0)	19 (17.3)	<0.0001
Left anterior descending artery	273 (66.6)	78 (47.0)	99 (73.9)	96 (87.3)	<0.0001
Left circumflex artery	186 (45.4)	56 (33.7)	67 (50.0)	63 (57.3)	<0.0001
Right coronary artery	203 (49.5)	70 (42.2)	62 (46.6)	71 (65.1)	<0.0001
Procedure					
Stents per patient	0.9 ± 1.1	0.7 ± 0.8	1.1 ± 1.0	1.0 ± 1.3	0.001
Total stent length per patient, mm	21.0 ± 25.5	15.1 ± 18.2	26.5 ± 25.3	23.1 ± 32.5	<0.0001
Residual SYNTAX Score	2.0 (0.0–8.0)	0.0 (0.0–4.0)	2.0 (0.0–9.5)	8.0 (0.0–15.0)	<0.0001
Physiological indexes					
FSSQFR	15.0 (5.0–22.0)	6.0 (2.0–8.0)	15.0 (15.0–17.5)	24.5 (22.3–28.3)	<0.0001
Residual FSSQFR	0.0 (0.0–7.0)	0.0 (0.0–0.0)	0.0 (0.0–9.0)	8.0 (0.0–17.0)	<0.0001
FFR/iFR	4 (1.0)	2 (1.2)	2 (1.4)	0 (0.0)	
Drug therapy					
Antiplatelets	247 (60.2)	106 (63.9)	77 (57.5)	64 (58.2)	0.465
ACE inhibitors	82 (20.0)	34 (20.5)	27 (20.1)	21 (19.1)	0.959
ARBs	124 (30.2)	57 (34.3)	41 (30.6)	26 (23.6)	0.165
b-blockers	188 (45.6)	73 (44.0)	62 (46.3)	53 (48.2)	0.775
Statins	206 (50.2)	89 (53.6)	65 (48.5)	52 (47.3)	0.521
Antidiabetics	80 (19.5)	30 (18.1)	23 (17.2)	27 (24.5)	0.292

Values are mean ± SD, median (ΙQR: 25–75%), or N (%). SYNTAX, Synergy Between Percutaneous Coronary Intervention with Taxus and Cardiac Surgery; QFR, quantitative flow ratio; FSSQFR, QFR-based functional SYNTAX Score; N, number of patients; PCI, percutaneous coronary intervention; STEMI, ST-segment–elevation myocardial infarction; NSTEMI, non-ST-segment–elevation myocardial infarction; CCS, chronic coronary syndrome; iFR, instantaneous wave-free ratio; FFR, fractional flow reserve; ACE, Angiotensin-converting enzyme; and ARBs, Angiotensin-receptors blockers.

**Table 2:** Outcomes overall and by FSSQFR groups.

	Overall (N = 410)	Low FSS (N = 166)	Intermediate FSS (N = 134)	High FSS (N = 110)	*p*-Value
Primary composite outcome	85 (20.7)	25 (15.1)	25 (18.7)	35 (31.8)	0.003
MACE	60 (14.6)	15 (9.0)	16 (11.9)	29 (26.4)	<0.0001
Cardiac death	28 (6.8)	4 (2.4)	10 (7.5)	14 (12.7)	0.004
Myocardial infarction	11 (2.8)	4 (2.5)	1 (0.8)	6 (5.7)	0.076
Ischemia-driven revascularization	21 (5.3)	7 (4.3)	5 (4.0)	9 (8.6)	0.224
Hospitalization for heart failure	10 (2.5)	2 (1.2)	5 (4.0)	3 (2.9)	0.326
Life-threatening arrhythmias	6 (1.5)	5 (3.0)	0 (0.0)	1 (0.9)	0.093
Angina					<0.0001
CCS I	204 (54.7)	91 (57.2)	64 (53.3)	49 (52.1)	
CCS II	55 (14.7)	14 (8.8)	19 (15.8)	22 (23.4)	
CCS III	10 (2.6)	0 (0.0)	1 (0.8)	9 (9.6)	
CCS IV	0 (0.0)	0 (0.0)	0 (0.0)	0 (0.0)	

Values are N (%). FSSQFR, QFR-based functional SYNTAX Score; N, number of patients; MACEs, major adverse cardiac events; CCS, Canadian Cardiovascular Society grading system.

## Data Availability

The data that support the findings of this study are available from the corresponding author upon reasonable request.

## Supplementary Materials

The following supporting information can be downloaded at: https://www.mdpi.com/article/10.3390/biomedicines12112437/s1, Figure S1: Event-free period by SS groups. Event-free curves depict (A) the composite primary endpoint; (B) MACEs; and (C) cardiac death. HRs and *p*-value referred to the high-risk group compared to the low-risk group. SS indicates Synergy Between Percutaneous Coronary Intervention with Taxus and Cardiac Surgery and MACEs indicates major cardiac adverse events; Figure S2: Correlation between FSSQFR and SS values; Figure S3: Receiver-operating characteristic curves of SS and FSSQFR for primary composite endpoint; Table S1: Reasons for excluding patients with non-analyzable QFR; Table S2: Univariate analysis of predictors for the primary outcome; Table S3: Baseline and Procedural Characteristics by SS Groups; Table S4: Baseline and lesion characteristics of reclassified patients based on FFSQFR; Table S5: Outcomes overall and by SS groups; Table S6: Outcomes by FSSQFR reclassification or not; Table S7: Outcomes by patients’ clinical presentation.

## References

[1] Neumann F.J., Sousa-Uva M., Ahlsson A., Alfonso F., Banning A.P., Benedetto U., Byrne R.A., Collet J.P., Falk V., Head S.J. (2019). 2018 ESC/EACTS Guidelines on myocardial revascularization. Eur. Heart J..

[2] Stone G.W., Kappetein A.P., Sabik J.F., Pocock S.J., Morice M.C., Puskas J., Kandzari D.E., Karmpaliotis D., Brown W.M., Lembo N.J. (2019). Five-Year Outcomes after PCI or CABG for Left Main Coronary Disease. N. Engl. J. Med..

[3] Holm N.R., Mäkikallio T., Lindsay M.M., Spence M.S., Erglis A., Menown I.B., Trovik T., Kalinauskas G., Mogensen L.J.H., Nielsen P.H. (2020). Percutaneous coronary angioplasty versus coronary artery bypass grafting in the treatment of unprotected left main stenosis: Updated 5-year outcomes from the randomised, non-inferiority NOBLE trial. Lancet.

[4] Ahn J.M., Roh J.H., Kim Y.H., Park D.W., Yun S.C., Lee P.H., Chang M., Park H.W., Lee S.W., Lee C.W. (2015). Randomized Trial of Stents Versus Bypass Surgery for Left Main Coronary Artery Disease: 5-Year Outcomes of the PRECOMBAT Study. J. Am. Coll. Cardiol..

[5] Serruys P.W., Morice M.C., Kappetein A.P., Colombo A., Holmes D.R., Mack M.J., Ståhle E., Feldman T.E., Van Den Brand M., Bass E.J. (2009). Percutaneous coronary intervention versus coronary-artery bypass grafting for severe coronary artery disease. N. Engl. J. Med..

[6] Fearon W.F., Nishi T., De Bruyne B., Boothroyd D.B., Barbato E., Tonino P., Jüni P., Pijls N.H.J., Hlatky M.A. (2018). Clinical outcomes and cost-effectiveness of fractional flow reserve-guided percutaneous coronary intervention in patients with stable coronary artery disease: Three-year follow-up of the FAME 2 trial (Fractional Flow Reserve versus Angiography for Multivessel Evaluation). Circulation.

[7] Xaplanteris P., Fournier S., Pijls N.H., Fearon W.F., Barbato E., Tonino P.A., Engstrøm T., Kääb S., Dambrink J.-H., Rioufol G. (2018). Five-Year Outcomes with PCI Guided by Fractional Flow Reserve. N. Engl. J. Med..

[8] van Nunen L.X., Zimmermann F.M., Tonino P.A.L., Barbato E., Baumbach A., Engstrøm T., Klauss V., A MacCarthy P., Manoharan G., Oldroyd K.G. (2015). Fractional flow reserve versus angiography for guidance of PCI in patients with multivessel coronary artery disease (FAME): 5-year follow-up of a randomised controlled trial. Lancet.

[9] Bech G.J.W., De Bruyne B., Pijls N.H.J., de Muinck E.D., Hoorntje J.C.A., Escaned J., Stella P.R., Boersma E., Bartunek J., Koolen J.J. (2001). Fractional flow reserve to determine the appropriateness of angioplasty in moderate coronary stenosis: A randomized trial. Circulation.

[10] Lim P.O. (2024). Is it a steal or a squeeze?. Hell. J. Cardiol. HJC.

[11] Adjedj J., De Bruyne B., Floré V., Di Gioia G., Ferrara A., Pellicano M., Toth G.G., Bartunek J., Vanderheyden M., Heyndrickx G.R. (2016). Significance of Intermediate Values of Fractional Flow Reserve in Patients with Coronary Artery Disease. Circulation.

[12] Nam C.W., Mangiacapra F., Entjes R., Chung I.S., Sels J.W., Tonino P.A., De Bruyne B., Pijls N.H., Fearon W.F. (2011). Functional SYNTAX score for risk assessment in multivessel coronary artery disease. J. Am. Coll. Cardiol..

[13] Terentes-Printzios D., Oikonomou D., Gkini K.P., Gardikioti V., Aznaouridis K., Dima I., Tsioufis K., Vlachopoulos C. (2022). Angiography-based estimation of coronary physiology: A frame is worth a thousand words. Trends Cardiovasc. Med..

[14] Choi K.H., Lee S.H., Lee J.M., Hwang D., Zhang J., Kim J., Im S.Y., Kim H.K., Nam C.-W., Doh J.H. (2021). Clinical relevance and prognostic implications of contrast quantitative flow ratio in patients with coronary artery disease. Int. J. Cardiol..

[15] Buono A., Mühlenhaus A., Schäfer T., Trieb A.-K., Schmeißer J., Koppe F., Münzel T., Anadol R., Gori T. (2020). QFR Predicts the Incidence of Long-Term Adverse Events in Patients with Suspected CAD: Feasibility and Reproducibility of the Method. J. Clin. Med..

[16] Vrints C., Andreotti F., Koskinas K.C., Rossello X., Adamo M., Ainslie J., Banning A.P., Budaj A., Buechel R.R., Chiariello G.A. (2024). 2024 ESC Guidelines for the management of chronic coronary syndromes. Eur. Heart. J..

[17] Zhang R., Song C., Guan C., Liu Q., Wang C., Xie L., Sun Z., Cai M., Zhang M., Wang H. (2020). Prognostic Value of Quantitative Flow Ratio Based Functional SYNTAX Score in Patients with Left Main or Multivessel Coronary Artery Disease. Circ. Cardiovasc. Interv..

[18] Vlachopoulos C., Dima I., Soulis D., Terentes-Printzios D., Skoumas I., Aznaouridis K., Solomou E., Richter D., Tousoulis D. (2020). Eligibility for PCSK-9 inhibitors treatment in acute coronary syndrome, chronic coronary artery disease and outpatient dyslipidemic patients. Atherosclerosis.

[19] Bundhun P.K., Sookharee Y., Bholee A., Huang F. (2017). Application of the SYNTAX score in interventional cardiology: A systematic review and meta-analysis. Medicine.

[20] Asano T., Katagiri Y., Chang C.C., Kogame N., Chichareon P., Takahashi K., Modolo R., Tenekecioglu E., Collet C., Jonker H. (2019). Angiography-Derived Fractional Flow Reserve in the SYNTAX II Trial: Feasibility, Diagnostic Performance of Quantitative Flow Ratio, and Clinical Prognostic Value of Functional SYNTAX Score Derived from Quantitative Flow Ratio in Patients with 3-Vessel Disease. Cardiovasc. Interv..

[21] Thygesen K., Alpert J.S., Jaffe A.S., Chaitman B.R., Bax J.J., Morrow D.A., White H.D. (2018). Fourth Universal Definition of Myocardial Infarction (2018). J. Am. Coll. Cardiol..

[22] Miyata K., Asano T., Saito A., Abe K., Tanigaki T., Hoshino M., Kobayashi T., Takaoka Y., Kanie T., Yamasaki M. (2022). Heart Team risk assessment with angiography-derived fractional flow reserve determining the optimal revascularization strategy in patients with multivessel disease: Trial design and rationale for the DECISION QFR randomized trial. Clin. Cardiol..

[23] Xu B., Tu S., Song L., Jin Z., Yu B., Fu G., Zhou Y., Wang J., Chen Y., Pu J. (2021). Angiographic quantitative flow ratio-guided coronary intervention (FAVOR III China): A multicentre, randomised, sham-controlled trial. Lancet.

[24] Pan J., Zhang Q., Lei L., Chen Y., Li G., Liang H., Lu J., Zhang X., Tang Y., Pu J. (2022). Impact of the caFFR-Guided Functional SYNTAX Score on Ventricular Tachycardia/Fibrillation Development in Patients with Acute Myocardial Infarction. Front. Cardiovasc. Med..

[25] Collison D., Copt S., Mizukami T., Collet C., McLaren R., Didagelos M., Aetesam-Ur-Rahman M., McCartney P., Ford T.J., Lindsay M. (2023). Angina After Percutaneous Coronary Intervention: Patient and Procedural Predictors. Circ. Cardiovasc. Interv..

[26] Panuccio G., Carabetta N., Torella D., De Rosa S. (2024). Clinical impact of coronary revascularization over medical treatment in chronic coronary syndromes: A systematic review and meta-analysis. Hell. J. Cardiol..

[27] De Luca L., Rosano G.M., Spoletini I. (2022). Post-percutaneous coronary intervention angina: From physiopathological mechanisms to individualized treatment. Cardiol. J..

[28] Ullrich H., Olschewski M., Belhadj K.-A., Münzel T., Gori T. (2022). Quantitative Flow Ratio or Angiography for the Assessment of Non-culprit Lesions in Acute Coronary Syndromes: Protocol of the Randomized Trial QUOMODO. Front. Cardiovasc. Med..

[29] Erbay A., Penzel L., Abdelwahed Y.S., Klotsche J., Heuberger A., Schatz A.-S., Steiner J., Haghikia A., Sinning D., Fröhlich G.M. (2021). Prognostic Impact of Pancoronary Quantitative Flow Ratio Assessment in Patients Undergoing Percutaneous Coronary Intervention for Acute Coronary Syndromes. Circ. Cardiovasc. Interv..

