# Supplementary material for: Prognostic Role of Functional SYNTAX Score Based on Quantitative Flow Ratio
Source: Biomedicines. 2024 Oct 24;12(11):2437. doi: 10.3390/biomedicines12112437 (PMC11592145; doi:10.3390/biomedicines12112437)
Supplement: Supplementary file 1 [file biomedicines-12-02437-s001.zip › biomedicines-3130519-supplementary.pdf]

Supplementary Material

Supplemental Figure S1. Event-free period by SS groups. Event-free curves depict (A) the composite primary endpoint; (B) MACEs and (C) cardiac death. HRs and p-value referred to the high-risk group compared to the low-risk group. SS indicates Synergy Between Percutaneous Coronary Intervention with Taxus and Cardiac Surgery and MACEs indicates major cardiac adverse events.

Supplemental Figure S2. Correlation between FSSQFR and SS values

Supplemental Figure S3. Receiver-Operating Characteristic Curves of SS and FSSQFR for Primary composite endpoint

Supplemental Table S1. Reasons for excluding patients with non-analyzable QFR

Supplemental Table S2. Univariate analysis of predictors for the primary outcome.

Supplemental Table S3. Baseline and Procedural Characteristics by SS Groups

Supplemental Table S4. Baseline and lesion characteristics of reclassified patients based on FFSQFR

Supplemental Table S5. Outcomes overall and by SS groups

Supplemental Table S6. Outcomes by FSSQFR reclassification or not

Supplemental Table S7. Outcomes by patients' clinical presentation

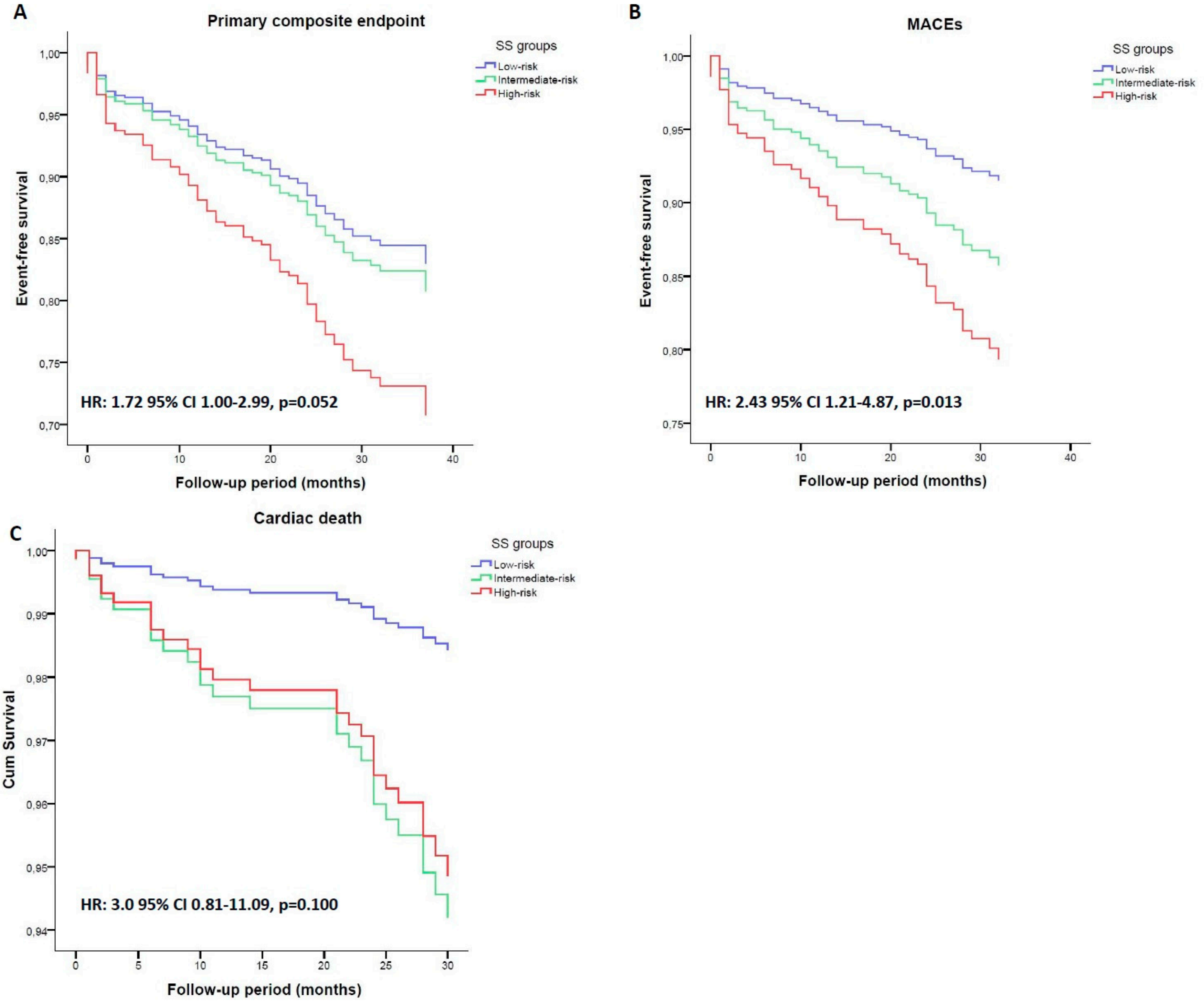

Figure S1. Event-free period by SS groups. Event-free curves depict (**A**) the composite primary endpoint; (**B**) MACEs and (**C**) cardiac death. SS indicates Synergy Between Percutaneous Coronary Intervention with Taxus and Cardiac Surgery and MACEs indicates major cardiac adverse events.

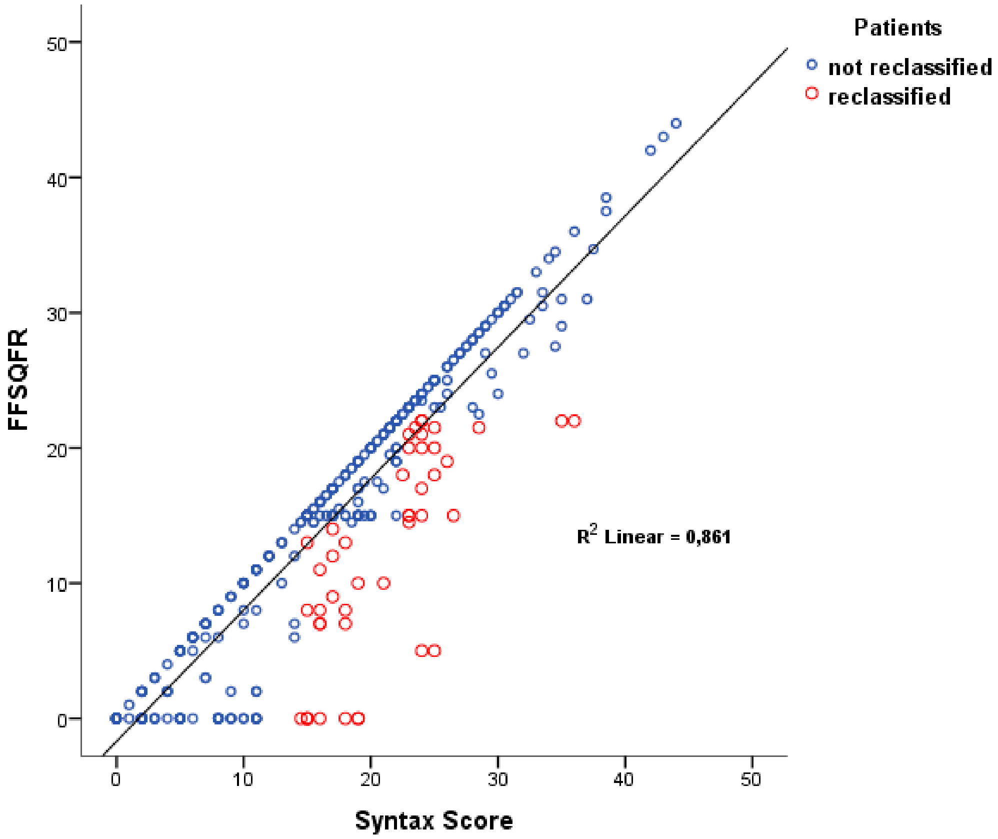

Figure S2: Correlation between FSSQFR and SS values

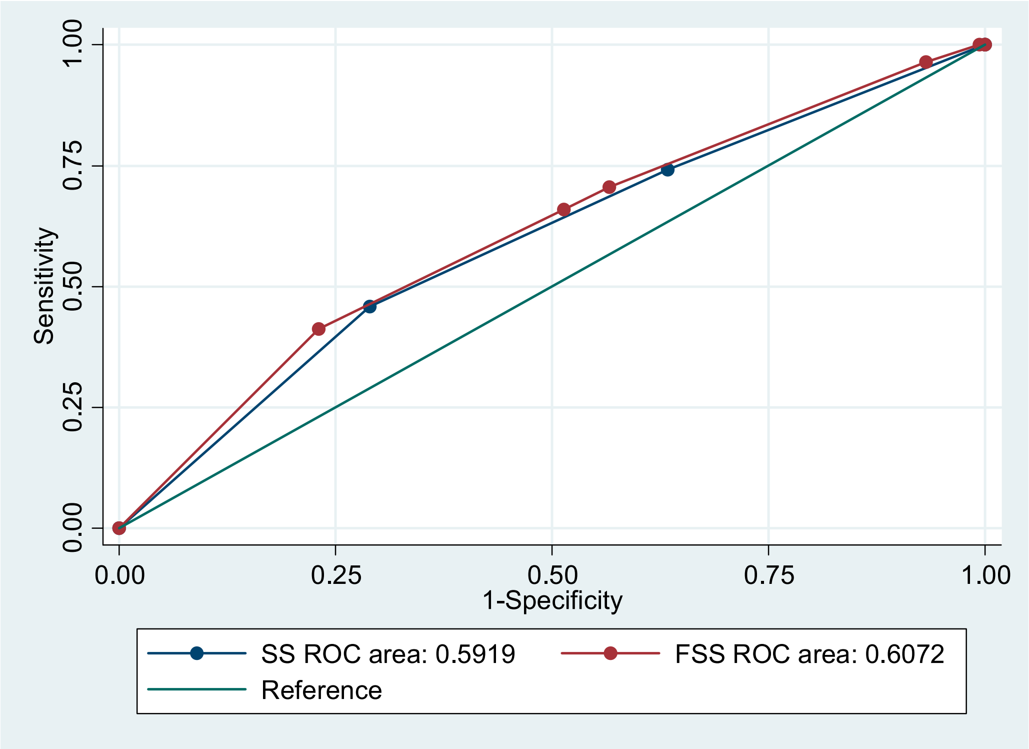

***p*=0.38 for comparison between the 2 curves.**

NRI=0.009, *p*=0.83 and IDI=0.0024, *p*=0.039

Figure S3: Receiver-Operating Characteristic Curves of SS and FSSQFR for Primary composite endpoint

Table S1. Reasons for excluding patients with non-analyzable QFR

	**Overall (N=1278)**
**Software incompatibility with coronary angiography**	423 (33.1%)
No isocenter data	320 (25%)
Lack of calibration data	103 (9%)
**Reasons for unsuccessful QFR computation**	150 (11.7%)
Absence of appropriate projections, n (%)	65 (5.1%)
Poor contrast opacification, n (%)	10 (0.7%)
Excessive vessel overlap and/or foreshortening, n (%)	7 (0.5%)
Small vessel (<2mm), n (%)	2 (0.2%)
Total occlusion, n (%)	6 (0.5%)
Bypass graft, n (%)	40 (3.1)
Aorto-ostial lesion, n (%)	20 (1.6)
**Patients with less than three analyzable QFR vessel**	295 (23%)

Table S2. Univariate analysis of predictors of the primary outcome.

**Variables**	**HR (95% CI)**	***p*-Value**
Age, year	1.023 (1.003-1.044)	0.026
Male	1.377 (0.794-2.250)	0.274
Body mass index, kg/m^2	0.961 (0.915-1.010)	0.114
Hypertension	1.629 (0.987-2.688)	0.056
Dyslipidemia	1.117 (0.713-1.945)	0.523
Smoking	1.284 (0.809-2.037)	0.289
Diabetes mellitus	1.707 (1.101-2.648)	0.017
Positive family history	1.260 (0.549-2.889)	0.586
Coronary Artery Disease	1.452 (0.943-2.237)	0.091
Previous myocardial infarction	1.522 (0.984-2.355)	0.059
Previous PCI	1.260 (0.786-2.021)	0.337
Previous stroke	1.946 (0.975-3.884)	0.059
Peripheral arterial disease	1.150 (0.466-2.841)	0.762
Heart Failure	3.209 (1.958-5.260)	<0.001
Ejection Fraction %	0.937 (0.917-0.958)	<0.001
STEMI	1.318 (0.783-2.218)	0.299
ACS	1.246 (0.810-1.917)	0.316
Left anterior descending artery	1.942 (1.165-3.236)	0.011
Stents per patient	0.990 (0.801-1.224)	0.925
Total stent length per patient, mm	0.997 (0.988-1.006)	0.528
Treatment_CABG	0.991 (0.478-2.052)	0.980
Conservative treatment	1.044 (0.620-1.757)	0.872
PCI_treatment	0.971 (0.618-1.528)	0.900
FSSQFR	1.031 (1.010-1.053)	0.004
SYNTAX score	1.039 (1.017-1.062)	0.001
Residual SYNTAX score	1.027 (0.987-1.070)	0.192
Residual FSS	1.053 (1.013-1.095)	0.009
b-blockers	0.648 (0.422-0.995)	0.048
ACE inhibitors	0.768 (0.607-0.973)	0.029
ARBs	0.656 (0.397-1.084)	0.100
Antidiabetics	0.657 (0.905-1.068)	0.090
Antiplatelets	0.912 (0.730-1.140)	0.420
Statins	0.768 (0.616-0.956)	0.018

PCI, Percutaneous coronary intervention; STEMI, ST-segment–elevation myocardial infarction; ACE, Angiotensin-converting enzyme; ARBs, Angiotensin receptor blockers; CABG, Coronary artery bypass graft; FSS_{QFR}, QFR-based functional SYNTAX score

Table S3. Baseline and Procedural Characteristics by SS Groups

	Low SS (N=141)	Intermediate SS (N=136)	High SS (N=133)	*p*-Value
**Clinical**				
Age, year	64.3±10.6	65.4±11.5	66.3±11.0	0.357
Male	105 (74.5)	116 (85.3)	119 (89.5)	0.003
Body mass index, kg/m^2	28.0±4.1	27.8±4.1	27.4±4.5	0.560
Hypertension	99 (70.2)	93 (68.4)	85 (63.9)	0.521
Dyslipidemia	111 (78.7)	112 (82.4)	104 (78.2)	0.650
Smoking	104 (73.8)	97 (71.3)	103 (77.4)	0.514
Diabetes mellitus	34 (24.1)	37 (27.2)	42 (31.6)	0.382
Positive family history	11 (7.8)	10 (7.4)	15 (11.3)	0.461
Coronary Artery Disease	41 (29.1)	44 (32.4)	48 (36.1)	0.464
Previous myocardial infarction	58 (41.1)	73 (53.7)	85 (63.9)	0.001
Previous PCI	37 (26.2)	30 (22.1)	30 (22.6)	0.669
Previous stroke	6 (4.3)	10 (7.4)	10 (7.5)	0.454
Peripheral arterial disease	8 (5.7)	7 (5.1)	6 (4.5)	0.909
Heart Failure	9 (6.4)	14 (10.3)	24 (18.0)	0.009
Ejection Fraction %	53.7±7.9	48.8±8.8	44.4±8.2	<0.0001
**Clinical presentation**				<0.0001
STEMI	11 (7.8)	26 (19.1)	38 (28.6)	
NSTEMI	16 (11.3)	23 (16.9)	26 (19.5)	
Unstable angina	11 (7.8)	4 (2.9)	5 (3.8)	
CCS	103 (73.0)	83 (61.0)	64 (48.1)	
**Angiographic**				
Baseline SYNTAX score	7.0 (5.0-10.0)	17.0 (15.0-18.5)	25.0 (22.4-28.1)	<0.0001
**Lesion location**				
Left main artery	1 (0.7)	2 (1.5)	21 (15.8)	<0.0001
Left anterior descending artery	61 (43.3)	97 (71.3)	115 (86.5)	<0.0001
Left circumflex artery	46 (32.6)	61 (44.9)	79 (59.4)	<0.0001
Right coronary artery	57 (40.4)	60 (44.4)	86 (65.2)	<0.0001
**Procedure**				
Stents per patient	0.6±0.7	1.1±1.0	1.0±1.3	<0.0001
Total stent length per patient, mm	14.2±17.4	26.0±24.6	23.1±31.5	<0.0001
Residual SYNTAX score	0.0 (0.0-2.0)	3.0 (0.0-8.8)	8 (0.0-17.0)	<0.0001
**Physiological indexes**				
FSS_{QFR}	6.0 (2.0-8.0)	15.0 (14.6-16.5)	23.5 (21.9-27.3)	<0.0001
Residual FSS_{QFR}	0.0 (0.0-0.0)	0.0 (0.0-6.8)	8 (0.0-16.4)	<0.0001
**Drug Therapy**				
Antiplatelets	88 (62.4)	87 (64.0)	72 (54.1)	0.208
ACE inhibitors	31 (22.0)	27 (19.9)	24 (18.0)	0.716
ARBs	46 (32.6)	45 (33.1)	33 (24.8)	0.252
b-blockers	63 (44.7)	65 (47.8)	60 (45.1)	0.855
Statins	73 (51.8)	73 (53.7)	60 (45.1)	0.337
Antidiabetics	25 (17.7)	23 (16.9)	32 (24.1)	0.270

Values are mean±SD, or median (IQR: 25%-75%), or n (%). FSS_{QFR}, QFR-based functional SYNTAX score; N, number of patients; PCI, percutaneous coronary intervention; STEMI, ST-segment–elevation myocardial infarction; NSTEMI, non–ST-segment–elevation myocardial infarction; CCS, chronic coronary syndrome; QFR, quantitative flow ratio; SYNTAX, Synergy Between Percutaneous Coronary Intervention With Taxus and Cardiac Surgery; ACE, Angiotensin-converting enzyme; and ARBs, Angiotensin-receptors blockers

Table S4: Baseline and lesion characteristics of reclassified patients based on FFSQFR

	Patients Not Reclassified (N=364)	Patients Reclassified (N=46)	*p*-Value
**Clinical**			
Age, year	65.0±10.9	67.6±11.7	0.143
Male	305 (83.8)	35 (76.1)	0.191
Body mass index, kg/m²	27.8±4.3	27.6±3.2	0.773
Hypertension	245 (67.3)	32 (69.6)	0.758
Dyslipidemia	291 (79.9)	36 (78.3)	0.789
Smoking	269 (73.9)	35 (76.1)	0.750
Diabetes mellitus	98 (26.9)	15 (32.6)	0.416
Positive family history	33 (9.1)	3 (6.5)	0.566
Coronary Artery Disease	120 (33.0)	13 (28.3)	0.521
Previous myocardial infarction	193 (53.0)	23 (50.0)	0.699
Previous PCI	90 (24.7)	7 (15.2)	0.153
Previous stroke	24 (6.6)	2 (4.3)	0.556
Peripheral arterial disease	19 (5.2)	2 (4.3)	0.800
Heart Failure	43 (11.8)	4 (8.7)	0.532
Ejection Fraction %	49.0±9.5	49.3±7.0	0.828
**Clinical presentation**			0.810
STEMI	68 (18.7)	7 (15.2)	
NSTEMI	59 (16.2)	6 (13.0)	
Unstable angina	17 (4.7)	3 (6.5)	
CCS	220 (60.4)	30 (65.2)	
**Angiographic**			
**Lesion location**			
Left main artery	22 (6.0)	2 (4.3)	0.644
Left anterior descending artery	239 (65.7)	34 (73.9)	0.263
Left circumflex artery	161 (44.2)	25 (54.3)	0.194
Right coronary artery	176 (48.6)	27 (58.7)	0.198
**Procedure**			
Stents per patient	0.9±1.1	0.9±1.0	0.838
Total stent length per patient, mm	21.1±25.7	21.6±23.9	0.875
**Drug Therapy**			
Antiplatelets	222 (61.0)	25 (54.3)	0.386
ACE inhibitors	76 (20.9)	6 (13.0)	0.211
ARBs	106 (29.1)	18 (39.1)	0.164
b-blockers	171 (47.0)	17 (37.0)	0.199
Statins	183 (50.3)	23 (50.0)	0.972
Antidiabetics	70 (19.2)	10 (21.7)	0.686

Values are mean±SD, or n (%). FSS_{QFR}, QFR-based functional SYNTAX score; N, number of patients; PCI, percutaneous coronary intervention; STEMI, ST-segment–elevation myocardial infarction; NSTEMI, non–ST-segment–elevation myocardial infarction; CCS, chronic coronary syndrome; ACE, Angiotensin-converting enzyme; and ARBs, Angiotensin-receptors blockers

Supplementary Table S5: Outcomes overall and by SS groups

	Overall (N=410)	Low SS (N=141)	Intermediate SS (N=136)	High SS (N=133)	*p*-Value
Primary composite outcome	85 (20.7)	22 (15.6)	24 (17.6)	39 (29.3)	0.011
MACE	60 (14.6)	12 (8.5)	17 (12.5)	31 (23.3)	0.002
Cardiac death	28 (6.8)	3 (2.1)	11 (8.1)	14 (10.5)	0.017
Myocardial infraction	11 (2.8)	3 (2.2)	2 (1.6)	6 (4.7)	0.269
Ischemia-driven revascularization	21 (5.3)	6 (4.3)	4 (3.1)	11 (8.7)	0.115
Hospitalization for heart failure	10 (2.5)	2 (1.4)	3 (2.3)	5 (3.9)	0.434
Life-threating arrythmias	6 (1.5)	5 (3.6)	0 (0.0)	1 (0.8)	0.039
**Angina symptoms**					<0.0001
CCS I	204 (54.7)	73 (54.1)	69 (57.0)	62 (53.0)	
CCS II	58 (15.3)	12 (8.9)	17 (14.0)	26 (22.2)	
CCS III	10 (2.6)	0 (0.0)	1 (0.8)	9 (7.7)	
CCS IV	0 (0.0)	0 (0.0)	0 (0.0)	0 (0.0)	

Values are n (%). SS, Syntax score; N, number of patients; MACE, major adverse cardiac events; CCS**,** Canadian Cardiovascular Society grading system

Table S6. Outcomes by FSSQFR reclassification or not

	Patients Not Reclassified (N=364)	Patients Reclassified (N=46)	*p*-Value
Primary composite outcome	78 (21.4)	7 (15.2)	0.328
MACE	55 (15.1)	5 (10.9)	0.443
Cardiac death	27 (7.4)	1 (2.2)	0.184
Myocardial infarction	10 (2.7)	1 (2.2)	0.807
Ischemia-driven revascularization	18 (4.9)	3 (6.6)	0.671
Hospitalization for heart failure	8 (2.2)	2 (4.3)	0.404
Life-threating arrythmias	6 (1.6)	0 (0.0)	0.371

Values are n (%). FSS_{QFR}, QFR-based functional Syntax score; N, number of patients; MACE, major adverse cardiac events

Table S7. Outcomes by patients' clinical presentation

	ACS (N=160)	CCS (N=250)	*p*-Value
Primary composite outcome	36 (22.5)	49 (19.6)	0.480
MACE	31 (19.4)	29 (11.6)	0.030
Cardiac death	17 (10.6)	11 (4.4)	0.015
Myocardial infraction	3 (1.9)	8 (3.3)	0.419
Ischemia-driven revascularization	11 (7.2)	10 (4.1)	0.190
Hospitalization for heart failure	2 (1.3)	8 (3.3)	0.224
Life-threating arrythmias	1 (0.7)	5 (2.1)	0.265

Values are n (%). N, number of patients; MACE, major adverse cardiac events
